# Exploring minimal models of sensory integration in nematode *C. elegans*

**DOI:** 10.1186/1471-2202-14-S1-P69

**Published:** 2013-07-08

**Authors:** Tom Sanders, Netta Cohen

**Affiliations:** 1 School of Computing, University of Leeds, Leeds, LS2 9JT, UK

## 

To survive, animals must select and execute appropriate actions in response to cues in rich, variable environments. Key to effective sensory processing is the ability to integrate inputs from different sensory modalities in a behaviorally meaningful way. Remarkably, even the simplest animals exhibit non-trivial forms of sensory integration. The roundworm *Caenorhabditis elegans *is a particularly interesting system in this context, due to the compactness of its small nervous system: unlike the modular organization of vertebrate or even insect brains, in *C. elegans*, sensory neurons responding in all different modalities (smell, taste, touch, temperature, and much more) all project onto a single circuit, the so called head navigation circuit. How this circuit integrates these inputs to generate appropriate behaviors is a wide open question. In fact, past computational models of this system short circuit the problem, by connecting model sensory neurons directly to motor neurons. That said, this approach may be adequate for modeling sensory responses to a single set of sensory neurons, and a single stimulus type. Here we take the first steps to model sensory integration, with the aim of better understanding the computational role of the navigation circuit and its constituent neurons.

We follow an iterative process of model design, validation and refinement against experimental data, and design and implementation of critical *in silico *experiments to test model predictions. We begin with a model, inspired by results from published sensory integration experiments [1], with a minimal set of neurons directly implicated in the computation. Importantly, this model includes sensory, interneuron and motor layers; it is embodied and fully situated, allowing us to test the model performance against experimentally obtained behavioral metrics. We explicitly demonstrate the necessity of including a full sensorimotor pathway to assess multi-sensory integration.

The motor behavior of the worm is relatively well characterized. However, this classification relies mostly on experiments in the absence of external stimuli, or during navigation on gradients of attractive chemicals. Our integration experiments are based on responses to a combination of attractive and repulsive stimuli. Interestingly, we find that the existing repertoire of behaviors fails to account for the animal's responses to repellents. Therefore, a key prediction of our model is the existence of a novel motor behavior mediated by a distinct sensorimotor pathway.

Surprisingly, we prove that a fully linear circuit, with rectified motor outputs is sufficient to qualitatively capture complex sensory integration as seen in behavioral data. This result is consistent with experimental findings implicating a single pair of interneurons in this form of sensory integration [1] but dispenses the postulated requirements for nonlinear input-output function of these neurons and feedback connections to the sensory layer. Furthermore, we show that changes in the ratios of afferent synaptic weights from different sensory neurons onto a linear integration interneuron allow precise control over the processing of single and combined stimuli. Implementation of novel sensory receptor properties and a sigmoidal activation function in the integration interneuron results in quantitatively correct model performance. Finally, we study the behavior of defective worms, (with virtually mutations or ablated neurons) and use this analysis to predict a role for additional interneurons in the navigation circuit.
